# The Mediterranean diet: a historical perspective

**DOI:** 10.1007/s40520-023-02686-3

**Published:** 2024-03-23

**Authors:** Antonio Capurso

**Affiliations:** Department of Internal Medicine and Geriatrics, School of Medicine, University of Bari, Bari, Italy

**Keywords:** Mediterranean diet, Romans, Barbarians, Arabs, Christopher Columbus

## Abstract

The Mediterranean diet, which was born in the Mediterranean basin, was initially quite poor and simple, essentially based on the products that grew almost spontaneously along the shores of the Mediterranean, i.e., olives, grapes, and wheat, which were long cultivated in the Mediterranean region. The invasions of the Roman Empire by barbarian populations, between 400 and 800 AD, made the diet enriched with products from wild uncultivated areas, meat from game and pigs, and vegetables. With the arrival of the Arabs in southern Italy in the ninth century, the focus of the diet shifted to carbohydrates, particularly to dried pasta and to other new ingredients. The Arabs primarily brought a new imaginative spirit to the kitchen by introducing and using an infinity of condiments and seasonings. The discovery of the Americas and the arrival of new ingredients from the New World brought the final adjustments to the Mediterranean diet: new meat (turkey), new vegetables (potatoes, broad beans, corn, tomatoes,) new fruits (strawberries, pineapples, coconuts, peanuts), chocolate, coffee and sugar completed the list of components of the Mediterranean diet as we know it today.

## The origins of the Mediterranean diet

The Mediterranean diet is foundamentally the dietary regimen that was followed in ancient times by the populations living along the Mediterranean coast, a place where olive trees and vignards grew spontaneously from time immemorial and olive oil was the main fat component. Other essential elements were subsequently added at later dates.

At the beginning, the original Mediterranean diet was based on the “*bread-olive oil-wine*” triad, integrated with legumes and cheeses produced with the milk of lean sheep and goat. Meat was eaten only infrequently, generally on very special occasions, such as during religious festivals, weddings, etc.

In that time, this essentially vegetarian diet was followed by the Greek and Roman populations. Rome had strong ties with the Greek culture which influenced profoundly the Roman life, its democracy, phylosophy, literature, language (the roman aristocrats spoke fluently Greek) and the alimentary habit. Cereal growing, olive tree and vine arboriculture were in fact the core elements underlying the productive system of the Mediterranean basin, where the Greco-Roman civilization was born and evolved.

The Mediterranean alimentary style was, of course, bound to progressively change over the time, in connection to the historical events that took place in Europe and Italy in the subsequent centuries [1–8] (Table 1).

**Table 1:** Historical stages of the Mediterranean diet

1. The earliest Mediterranean diet
2. The clash with barbarian culture
3. The influence of the Arabs
4. The discovery of the Americas

## The fall of the Roman Empire and the barbarians

When the last Roman Emperor Romulus Augustulus was deposed, in 476 AD, the Roman Empire was already heavily undermined by infighting and by complex internal political events. From that time, it continued to gradually dissolve as barbarian hordes, from the north, invaded the empire’s territories, bringing with them their brand of violence, as well as their culture and eating habits.

It should be specified that “*barbarians*”, in the Latin, simply meant “foreigners”.

The previous sack of Rome, which occurred in 410 AD, undertaken by the Visigoths led by Alaric, was decisive for the subsequent fall of the Roman Empire. It was in fact a dramatic event that led to the Empire’s moral collapse and set the stage for its final dissolution.

The date of 476 AD is historically the watershed between the end of the “*Antiquity Era*” and the “*Middle Ages*”.

Over the following centuries, between 400 and 800 AD, sizable “b*arbarians*” tribes essentially of Celtic and Germanic origin, migrated from the north-east towards the south of Europe and to the epicentre of the dissolved Roman Empire. The Lombards, who reigned in Italy from 568 to 774 AD, and the Franks of Charlemagne, who defeated the Lombards and reigned from 774 to 814 AD, were the two “barbarian” populations who played the main role in estabilishing the new cultural and alimentary habits in those areas.

## The historical scenario around 1000 AD: the impact of “*barbarian*” culture

As a consequence of these historical events, at about 1000 AD the alimentary habits of the Mediterranean populations reflected those of two well defined models of civilizations: the “*classical*” Greco-Roman one and the “*barbarian*” Celtic and Germanic one, two very different civilizations that had clashed for quite some time.

The “*classical*” Greco-Roman civilization, which originated and evolved in the Mediterranean basin, was primarily based on cereal (wheat) production and arboriculture (olive trees and vineyards), and rearing sheep and goats. This productive model and food culture was firmly linked to the “*bread-olive oil-wine*” triad, integrated with legumes and sheep–goat cheeses.

The “*barbarian*” Celtic-Germanic civilization, inhabiting wild, forested areas of the continental Europe, had developed by semi-nomadic populations that hunt, fished, gatheed wild fruit, and reared free-range livestock, mostly pigs. Their economy was a silvo-pastoral one, entirely different from the Greco-Roman triad, the “barbarian” version essentially based on the “*meat-lard-beer*” triad. In this context, the cereal growing production was a marginal activity, in that it was grown to be used to produce beer, not flour for bread production.

**Fig. 1:**
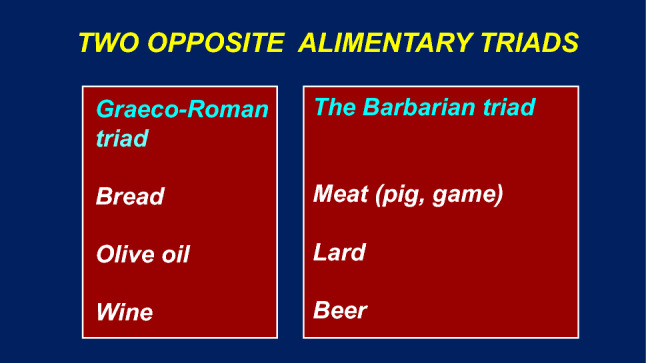
The different alimentary triads of *Graeco-Roman* and *Barbarian* populations, the former essentially vegetarian, the latter of animal origin

The two dietary models were, therefore, conceptually very different from one another, as they hinged on two very different conceptual entities (Fig. 1).

The “*classical*” Greco-Roman cultural model was based on a mind–body holism*,* that sought physical pleasure and well-being via a balance between mental and physical health, the latter connected to the dietary notion of the body regimen, outlined by Hippocrates in his big work “*Corpus Hippocraticum*” [9].

The “b*arbarian*” Celtic-Germanic model, introduced by the Lombard and the Francks was, on the contrary, strongly focused on physical strenght and skill vigor, courage and vitality, all qualities that associated to their characteristics, bellicosity and fearlessness which were prized by the Celtic-Germanic warrios aristocrats. From the Greek-Roman perspective, they were primitive uncivilized men who were capable of consuming huge amounts of food and beverages.

Those two very different cultural models and entities came to clash with one another as each civilization tried to forcefìully impose itself on the other. Eventually they blended together and begot a new enriched dietary regimen.

Briefly then, this is how the practice of growing cereals grapes, and olives typical of the Mediterranean civilization spread to the north, and likewise how bread, wine, and oil, the basic elements of the Mediterranean diet, became widespread in northern European countries, mostly in connection to their role in the rituals and symbols of Christianity. In fact, it was particularly through churches and monasteries that the food model of the “*classical*” civilization spread to the north, although these products did not remain confined to the liturgical sphere but rapidly affected people’s daily food habits.

While the Mediterranean diet was travelling to the north, the Celtic and Germanic productive model, together with its political and social institutions progressively spread to the south of Europe. As a result, a different view towards uncultivated areas, woods, pasture, and marshes, all present in the south but practically untouched during the pre-medioeval periodd, developed in the southern regions, particularly in the Southern Italy This shift in the peasant’s conservative attitude led to the diffusion of a mixed “agro-silvo-pastoral” system in the south based on a new fooding model in which animal products played a more important role.

But, while the northern italian regions, particularly Emilia, who quickly became Germanized adopting models incorporating large quantities of meat (especially pork), lard and beer, the Southern Italian regions substantially continued to produce grain, olive oil and wine, the typical products of the “*classical*” Roman-Mediterranean civilization. This resistance to innovations was linked to the peasants’ conservative attitude towards their traditions and their dislike of change. Eventually they ended up accepting some of the “innovations” the “*barbarian*” culture brought. Given the abundance of marshes, ponds, rivers, streams, and lakes in Southern Italy, the populations living there began to exploit those resources. As a result, freshwater fish consumption began to spread extensively and to take its place alongside sea fish consumption. The people started to exploit the wild uncultivated areas which became sites for breeding, hunting and harvesting wild fruits. This development led to an improved, enriched diet, which was still essentially based on the “*bread, olive oil and wine*” triad. Finally, the southern peasants adopted the “barbarianas’ “ practice of growing vegetables in small plots of land behind their homes, and thus introducing new vegetables into their daily diet.

## The arrival of the Arabs’ culture

A further enrichment of the Meditarrenean diet occurred when the Arabs arrived in Southern Italy, in the ninth century.

After conquering the north-African Mediterranean countries, in 711 AD the Arabs crossed the Strait of Gibraltar and over the course of the next 20 years they conquered Spain and South-western regions of France. In 827 AD, the Arabs reached Sicily and conquered the island in only 4 years time and extended their dominion to part of Southern Italy. The Arabs remained in Sicily until 1091 AD, when they were defeated by the Norman Roger II, of the house of Altavilla.

Unlike the Saracen militias, who were a violent people from the Maghreb region, the Arabs possessed a very high level of education and knowledgeable about mathematical, literary, phylosophical, geographic and agronomic topics. Their arrival in Sicily, therefore, brought about important cultural improvements and, above all, added many new ingredients and recipes to the people’s ordinary diet.

Compared to the “*classical*” Greco.Roman and the “*barbarian*” dietary style, the Arab food model had many more ingredients and recipes, to maximize tastes and flavours. Thus, while the “*classical*” food culture was essentially vegetarian and the “*barbarian”* one was based principally on meat and lard, the Arabs food culture focused on carbohydrates, particularly on pasta, rice and sugar and, as a result, these elements quickly entered the Mediterranean food culture and became staples in the people diets. The Arabs also introduced many new completely unknown fruits and vegetables, such as eggplants, artichokes, spinachs, limes, bananas, lemons, oranges, as well as many spices, such as cinnamom, cloves, nutmeg, ginger, and saffron.

## The introduction of dried pasta and rice by the Arabs

Dried pasta was perhaps the most important innovation introduced by the Arabs to the Italian food culture. Fresh homemade pasta was well known in ancient Rome, but it played only a marginal role in the culinary habits of the Roman population, probably because it was unappealing due to the poverty of the condiments that were utilized. In fact, after boiling in water (sometimes in rose water) or in milk, they seasoned the fresh-made pasta with grated cheese or chopped almonds, sometimes with the “*Garum”,* (also called “*Liquamen”)*, an anchovy sauce flavoured with spices.

The dried product introduced by Arabs was instead long lasting and portable, and therefore suitable for commerce and exportation. To produce the dry pasta on a large scale, the Arabs had set up a mill in a small town 40 km away from Palermo, Trabia, where they had found fresh water and the favorable climatic conditions (windy) needed for drying pasta.

Arabs also helped to spread knowledge about pasta as a staple food. They in fact favoured its diffusion by showing how to mix the pasta with an infinite variety of ingredients, to enhance its flavour and taste. The Arabs had learned to mix the pasta with greens (vegetables), legumes, fish, seafood, minced meat (i.e., the “*lasagna*” or the “*timbale*”, a pasta seasoned with cheese, small meat balls, eggs and other ingredients), and with a number of spices.

Importantly, the Arabs also introduced rice, which since ever had been a staple of Arabs’ cuisine. The Arabs used rice as a substutution for bread and it was generally served mixed with other foods, generally meat, vegetables and/or sea foods. It was also mixed with other ingredients, as in the “*rice timbale*” dish, or fried, as in the case of fried rice-balls (the balls are made with rice covering a peace of cheese or a small portion of minced meat), or finally used in the “*risotto*”, a rice slowly cooked with seafood or vegetables and seasoned with spices.

## The Arabs’ cane sugar

The introduction of cane sugar to a region where honey had been used exclusively until that time was another important innovation linked to the Arabs. When cane sugar became available, confections and sweets became even more popular. Of course, the Arabs introduced their typical candies, i.e., the “*cassata*” and the “*cannoli*” (a cylinder of fried tough stuffed with sweetened “*ricotta*”), as well as the marzipan, sesame nougat, cakes with custard, “*marron glacés*”, and many other confections. Fried or baked sweets stuffed with cream or other sweet ingredients, and also a forerunner of the modern ice cream, the lemon “*sharbet*”, which later became the “*sorbet*” were developed.

## The discovery of the America and the arrival of food products from the New World

In 1492, Christopher Columbus (known in Spain as Cristòbal Colòn) landed with three small ships, or caravels, in a small island of central America, the Bahamian island of Samana Cay, which Columbus named San Salvador. Three weeks later, the three-vessel fleet landed in Cuba, which they called Juana, and then in Hispaniola, today called Dominican Repubblic and Haiti. When Columbus landed in San Salvador, he encountered a thriving indigenous people, the Taino (a tribe of the native Arawaks) who drew sustenance from colorful native crops, fish, and game. Maize (corn), beans, squash and seafoods were the central components of their diet. Fish and turkey were the main sources of animal proteins. Other regional crops were potatoes, tomatoes, chili peppers, cassava, pumpkins and peanuts. Tropical fruit, such as pineapple, avocado, guava, and papaya enriched the native diet. These foods were entirely new and unfamiliar to Columbus and to his crew. Of course, they took as many of these new products as they could to Spain, when they returned, and these ended up becoming important components of the European diet.

It is important to remember that at the time of the America’s discovery the Europe had experienced several epidemics that had decimated the population, and also a “*little glaciation”* that occurred in the early fourtheenth century [10], all events that led to a significant contraction in cereal production and the onset of a terrible famine, aggravated by many wars that devastated the European continent in the fifteenth century.

At that time, the diet of most Europeans was based essentiallty on bread (up to 1.5 kg per person per day), pulses (chickpeas, peas, lentils, fava beans), goat and sheep cheeses, eggs, and rarely meat (pig, sheep, chickens) or fish (salted or dried). Game, resulting from poaching, was rather rare since only nobles were allowed to hunt. Wheat was frequently substituted by other cereals such as rye and oats, in the northern regions, and barley, millet and spelt in the southern ones. Lard and butter, in the north, and olive oil in the south, were used to season when they were available. Salt was rarely used because it was so expensive.

The new food products imported from the Americas were of foundamental importance for the European population as the existing cereal production was inadequate and the food culture excessively conservative. Potatoes, maize, broad beans, peppers, tomatoes, peanuts, pumpkin, pineapple, avocado quickly spread throughout Europe and the American turkey became a substitute for partridges, hares, peacocks and cranes.

However, not all of these new products were immediately used in the kitchen. Initially, in fact, many people were fearfull about eating potatoes as the rumor had spread that eating green potatoes, which contains solanin, a toxic alkaloid, caused severe gastro-enteric disorders. Later, however, potatoes became a part of people’s daily diet when they learned how to use them at the proper stage of growth.

Likewise corn (maize) initially met with quite a bit of distrust. Imported from Central America, it made its journey through Portugal, southern France, Spain, India, Mesopotamia and Turkey, finally endig up in Venice (the Venetians called it “*turkish grain*”). Although it was much more productive than wheat and could provide two yearly harvests, the idea of using it for human consumption encountered a great deal of resistance over a long period of time. In fact, it long remained a crop that was fed to cattle, pigs, ox, chickens and pigeon. Human beings generally ate corn only during times of famine. It was only in the XVIII century that corn became popular and a staple food for the lower classes. An almost exclusive consumption of corn by many peasants led, however, to an epidemic of “*pellagra*”, a disease linked to a deficiency of vitamin PP (vitamin B3, or niacin) that caused many deaths in northern areas of Italy up to the beginning of ‘900 s. While this vitamin is present in corn, the cooking method used by Europeans, i.e., simply boiling in hot water, did not allow its abrorption. The indigenous people of the America who had always eaten maize but never suffered from “pellagra”, in fact, cooked it in an alkaline solution (water with carbonate) which rendered vitamin PP bioavailable and absosbable.

Broad beans, on the contrary, were immediately popular and rapidly spread all over Europe. A variety of broad beans, the *cowpeas*, imported from Africa since 2000 b.C., were available in ancient Rome. They did not, however, play an important role in the diet of the Romans probably because they were not very tasty. The Mexican–American broad beans, on the contrary, were immediately a great success. They were bigger and tasted so much better than *cowpeas* and, importantly, they were easily cultivated in Europe’s temperate climates and could cheaply feed many hungry mouths.

Tomatoes found quite a bit of resistance at the beginning and needed quite a bit of time before they were accepted by the European and Italian cooks. Initially, they were considered ornamental plant that were possibly poisonous, given their brilliant red color. According one medioeval botany books, tomatoes can be eaten “*seasoned with pepper, salt and olive oil, but they give little and bad nourishment*” [11]. For two centuries, therefore, tomatoes were not at all popular and only at the end of 1700s they begin to appear in some cookery textbooks as an eccentricity not particlarly suitable for common use. Only at the end of 1800s tomatoes begin to spread and to become popular eventually becoming one of the main dressings for pasta and in particular for “spaghetti”.

Bell peppers were one of the first plants Columbus brought with him from the New World. Originally they were considered decorative plants. Later, Columbus also brought the chili peppers to Europe, an ingredient widely used by native Americans to season their food. In Europe, it was initially considered a spice. Its rapid diffusion was favoured both by the fact that it thrived in Europe’s temperate climate, and by its low cost compared to that of traditional spices. Its wide success was also due to the medicinal virtues of the active substance present in chili, the *capsaicin*, that has both anti-rheumatic and revulsive properties.

Cocoa and chocolate deserve a special mention. Cocoa seeds arrived in Europe in 1502, with the fourth and last trip of Columbus to the America, When Columbus landed in Honduras, the natives offered him a cocoa drink. On his return to Spain, he presented the cocoa seeds to the King, but no one was particularly impressed because the drink must have been very bitter. In 1528, Fernando Cortez presented King Charles V of Spain cocoa seeds. Liquid chocolate arrived for the first time in Siviglia in 1585 and in 1590 the bishop Francisco Juan Zumàrraga added some sugar and vanilla to the warm liquid chocolate, making it sweet and very appealing; from that time on, it was enthusiastically appreciated by many Europeans, although it remained for the most part known and loved in Spain. Only several years later the Spaniards exported chocolate to Modica, a Spanish protectorate in Sicily. There they taught the method of transforming chocolate into solid tablets, a procedure they had learned from Mexican natives. The Aztecs had called the chocolate paste that they obtained from minced seeds “xocoàti”, hence the name “chocolate”. Thereafter, chocolate quickly spreads to France, Germany, Switzerland and Italy. Numerous chocolate shops arose, first in Florence, then in Vernice, where chocolate quickly conquered the palate of the nobles. It is said that madame de Manteinon, the secret wife of Louis the XIV, King of France, could not live without chocolate. Likewise, supposedly Marie-Antoinette, the queen consort of King Louis the XVI, always traveled with her personal “*maitre chocolatier*”. Voltaire drank a dozen of cups a day to fight, he said, the weakness of the old age. Stendhal, Mozart, Goethe also loved chocolate.

### Other American fruits and vegetables

In addition to potatoes, corn, tomatoes, and beans, other important foods as courgettes, pumpkins, strawberries, pineapples, prickly pears, peanuts, sunflowers, coconuts, vanilla, cinnamon, cloves, coriander, curry, and paprika travelled to Europe.

## Additional benefits coming from the New World

On his second trip to America in 1493, Columbus brought some sugarcane plants from the Canary Islands with him. Soon after, sugarcane plantations were developed in Central America, due to the local temperate climate. Plantations were quickly developed in Puerto Rico, Cuba and in Jamaica. In a short time, much of the New World was covered with sugarcane plantations, thus favoring the production and the exportation of cane sugar to Europe and, above all, making sugar cheap and accessible to most people. While sugar was already present in Europe since about 900, when it was imported by the Arabs to Spain and Sicily, its diffusion was for the most part confined to Andalusia and Sicily due to its limited production.

As far as the coffee is concerned, the French Navy officer, *Gabriel de Clieu*, set sail in 1720 for the Caribbean, bringing two small coffee plants with him. Coffee plantations quickly spread throughout Central America, arriving in Santo Domingo in 1725, in Guadaloupe in 1726, in Giamaica in 1730, in Cuba in 1748, and finally in Puerto Rico in 1755.

At more or less the same time, In 1718, the Dutch brought coffee plants to the Dutch Guiana. Coffee plants were then taken to French Guiana two years later and finally to Brazil in 1727, where large coffee plantations quickly arose. Coffee plantations required intensive manual labor and thus led to the development of slavery and the transportation of human beings who were captured in Africa and forcibly deported to the New World.

## Conclusions

The Mediterranean dieti was originally very sparing and based on “*bread-wine-olive oil*” triad, integrated with some legumes and cheeses. This very poor alimentary style was bound to progressively change over the time, following the historical events that took place in Europe and Italy in the subsequent centuries. In this way, the diet progressively changed enriching with new products and new recipes and gradually reaching its definitive profile, the current one.

## Data Availability

No data set was used for the research described in the article.
